# Stability, Antioxidant Activity and Intestinal Permeation of Oleuropein Inclusion Complexes with Beta-Cyclodextrin and Hydroxypropyl-Beta-Cyclodextrin

**DOI:** 10.3390/molecules27165077

**Published:** 2022-08-10

**Authors:** Hui Liu, Jinhua Luo, Ping Yang, Xiulan Yang, Jun Yan, Qian Yao

**Affiliations:** 1 Key Laboratory of Medicinal and Edible Plants Resources Development of Sichuan Education Department, Sichuan Industrial Institute of Antibiotics, School of Pharmacy, Chengdu University, Chengdu 610106, China; 2 School of Food and Bioengineering, Chengdu University, Chengdu 610106, China

**Keywords:** oleuropein, beta-CD, hydroxypropyl-beta-CD, stability, intestinal permeation

## Abstract

Compared to beta-cyclodextrins (beta-CD), hydroxypropyl-beta-cyclodextrins (HP-beta-CD) are a more popular material used to prepare inclusion complexes due to their superior solubility and intestinal absorption. In this study, oleuropein (OL) inclusion complexes with beta-CD (beta-CD:OL) and HP-beta-CD (HP-beta-CD:OL) were prepared and the formation of inclusion complexes was validated by IR, PXRD, and DSC. A phase solubility test showed that the lg*K* (25 °C) and binding energy of beta-CD:OL and HP-beta-CD:OL was 2.32 versus 1.98, and −6.1 versus −24.66 KJ/mol, respectively. Beta-CD:OL exhibited a more powerful effect than HP-beta-CD:OL in protecting OL from degradation upon exposure to light, high temperature and high humidity. Molecular docking, peak intensity of carbonyls in IR, and ferric reducing power revealed that beta-CD:OL formed more hydrogen bonds with the unstable groups of OL. Both inclusion complexes significantly enhanced the solubility, intestinal permeation and antioxidant activity of OL (*p* < 0.05). Though HP-beta-CD:OL had higher solubility and intestinal absorption over beta-CD:OL, the difference was not significant (*p* > 0.05). The study implies that lower binding energy is not always associated with the higher stability of a complex. Beta-CD can protect a multiple-hydroxyl compound more efficiently than HP-beta-CD with the intestinal permeation comparable to HP-beta-CD complex.

## 1. Introduction

**Figure 1:**
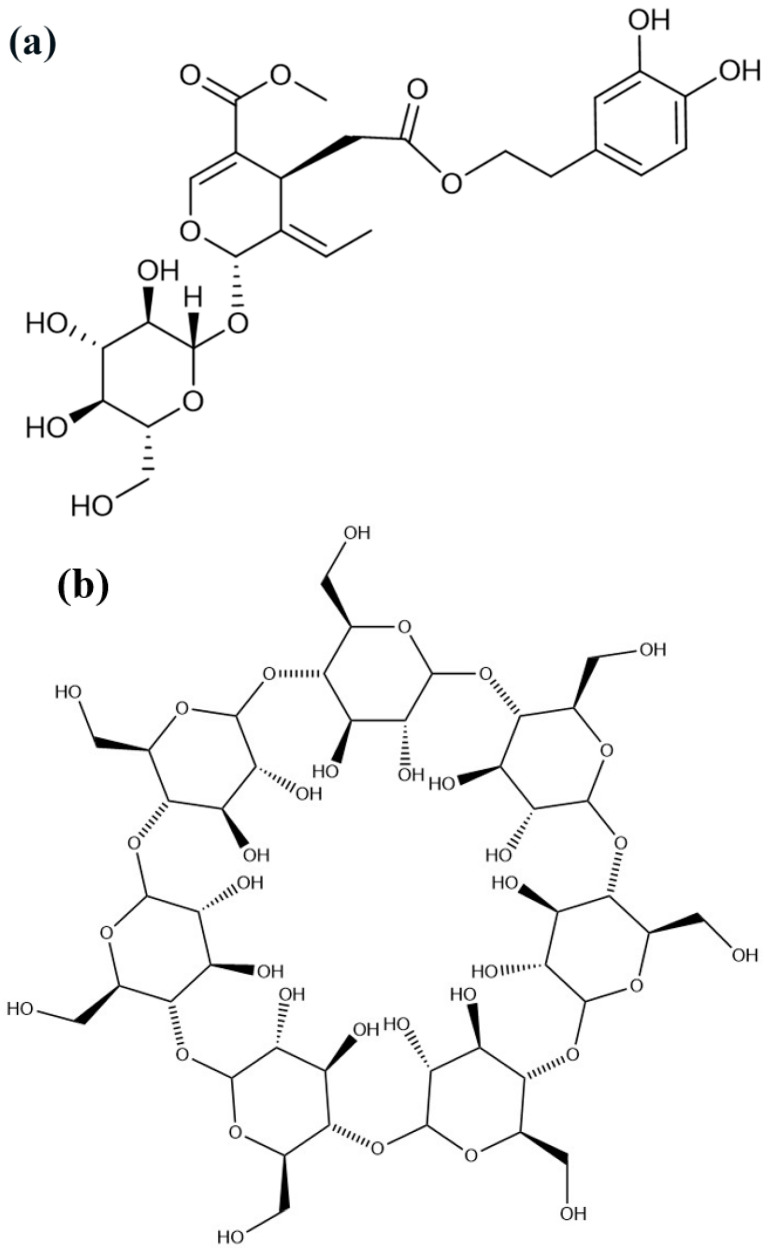

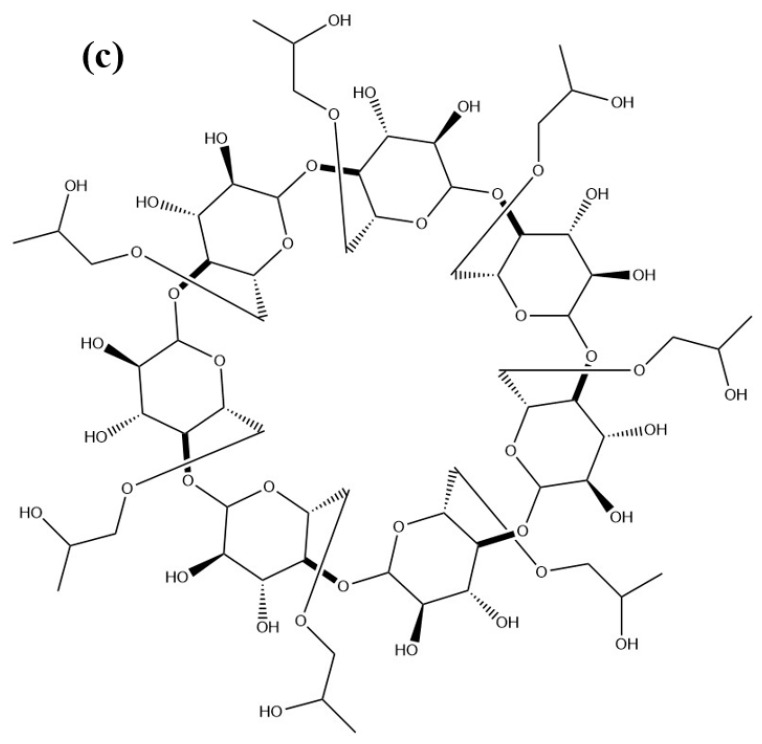
Chemical structures of OL (**a**), beta-CD (**b**), and HP-beta-CD (**c**). OL, oleuropein; beta-CD, beta-cyclodextrins; HP-beta-CD, hydroxypropyl-beta-cyclodextrins.

Oleuropein (OL, Figure 1a) is the major phenolic compound extracted from olive leaves. It is also present in olive fruits and virgin olive oil and is responsible for the characteristic bitter taste of unprocessed olives. OL exhibits numerous biological and pharmacological activities, including antioxidant [1], anti-inflammatory [2], anti-apoptotic functions [3], and so on [4]. In addition, OL was efficient in inhibiting hyperglycemia and oxidative stress induced by diabetes, suggesting that OL may help to prevent oxidative stress-related diabetes complications [5]. However, due to the phenol hydroxyls and ester bonds present in the structure, OL is unstable under high temperature and humidity. In addition, the bitter taste prevents its application in oral administration. How to improve the stability of OL and reduce its irritation of the gastrointestinal tract, has become the major concern regarding OL utility.

**Figure 2:**
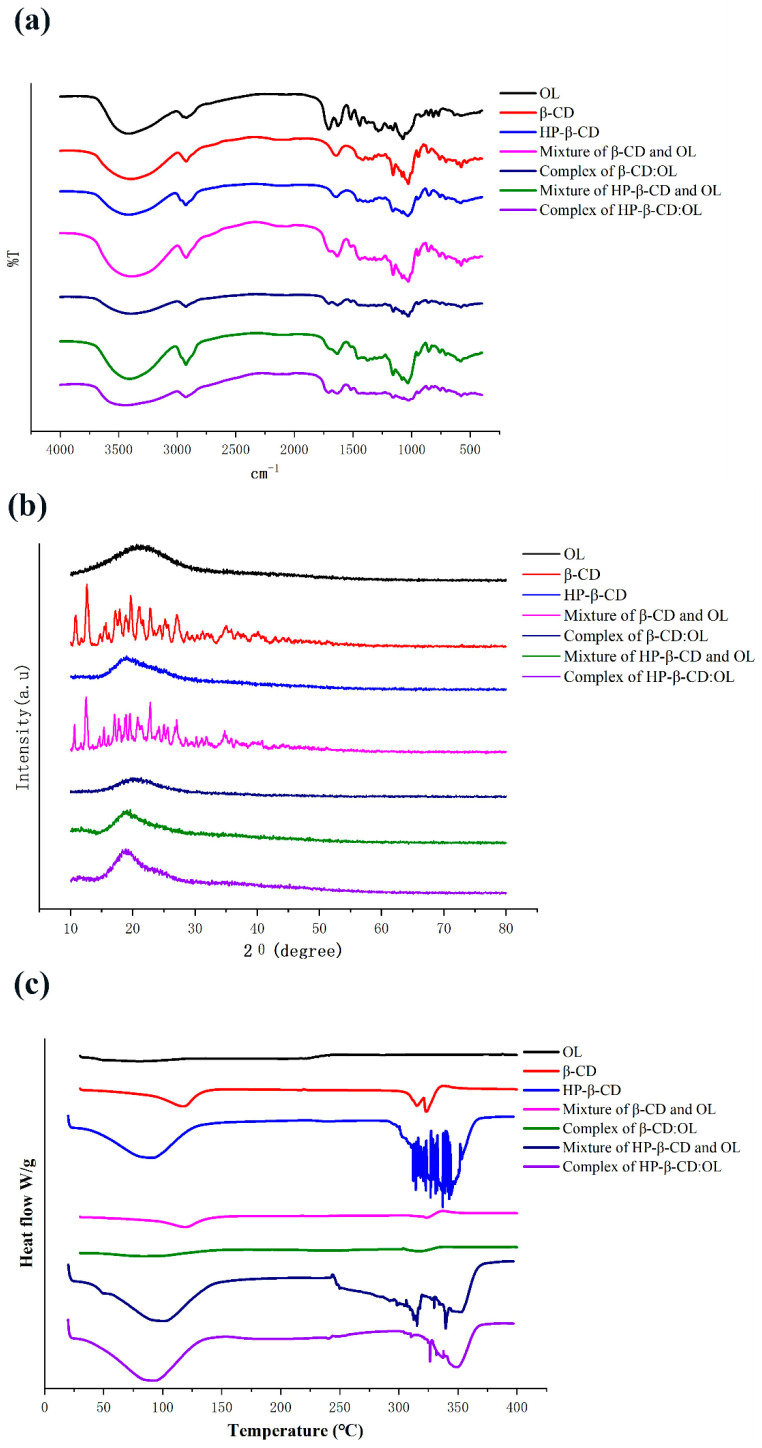
IR spectra (**a**), PXRD profiles (**b**), and DSC thermograms (**c**) of OL, beta-CD, HP-beta-CD, the physical mixture of beta-CD and OL, inclusion complex beta-CD:OL, the physical mixture of HP-beta-CD and OL, and inclusion complex HP-beta-CD:OL. The abbreviations are as Figure 1.

Cyclodextrins (CD) are a series of cyclic oligosaccharides yielded from the enzymatic degradation of starch [6]. Among them, beta-cyclodextrin (beta-CD, Figure 1b) and 2-hydroxypropyl-beta-cyclodextrin (HP-beta-CD, Figure 1c) are the most used. The hydrophobic cavity of CD provides a classical binding site for hydrophobic molecules [7]. Active components can completely or partially enter the lipophilic cavity of CD through hydrophobic, electrostatic, Van der Waals interactions, or hydrogen bonding, and construct a structure with high stability. CD and their derivatives can also improve the solubility of insoluble compounds by encapsulating guest molecules in its cavity [6]. Moreover, the inclusion complex with CD has many other useful properties, for example, to reduce irritation, toxicity and side effects, modulate drug release, enhance the bioavailability [8], and reduce the unpleasant smell and taste emitted by the component itself [9].

The solubility of the HP-beta-CD complex is usually higher than that of the beta-CD complex owing to the reduced interaction among HP-beta-CD molecules and the resultant enhanced affinity with water molecules by modifying hydroxyls into hydroxypropyls [10]. This attribute of HP-beta-CD makes it more popular in the preparation of CD-based inclusion complexes. In this study, OL inclusion complexes with beta-CD and HP-beta-CD were prepared (beta-CD:OL and HP-beta-CD:OL), respectively, in an attempt to enhance the stability and reduce the irritation of OL. The stability, antioxidant activity, solubility and intestinal permeation of the two complexes were studied comprehensively to clarify which material is more suitable for OL, a compound with multiple hydroxyls and ester groups.

## 2. Results and Discussion

### 2.1. Encapsulation Efficiency and Drug Loading

The encapsulation efficiency (EE) of beta-CD:OL and HP-beta-CD:OL was (85.56 ± 0.24)% and (94.21 ± 0.17)%, respectively. The drug loading (DL) was (29.96 ± 0.37)% and (26.23 ± 0.34)%, respectively.

### 2.2. Confirming the Formation of Inclusion Complex

#### 2.2.1. Fourier-Transform Infrared (FT-IR) Spectroscopy

The IR spectra of individual compositions and inclusion complexes are shown in Figure 2a. The spectrum of OL includes the peaks of 3425, 2924, 1710, and 1076 cm^−1^, which align with the stretching vibration of -OH, -CH_2_, -C = O, and C-O-C, respectively. The characteristic peaks of -OH, -CH_2_, -C = O, and C-O-C also appear in the spectra of beta-CD and HP-beta-CD. Furthermore, they have another peculiar peak of 1160 cm^−1^, which is derived from the stretching vibration of C-O-C of the glycosidic bond. The physical mixture of OL with beta-CD or HP-beta-CD maintain all of the characteristic peaks of the two compounds with obviously increased intensity. In contrast, compared to the individual compounds, all of the characteristic peaks of inclusion complexes, like -OH, C-O-C and -C = O, become smaller, which may be attributed to the formation of a hydrogen bond between OL and CD [11,12]. For beta-CD:OL, the peak of -C = O from OL is too small to be noticed. The -OH peak of HP-beta-CD:OL presents an evident red shift and dwindling. The changes in peak position and intensity indicate the successful formation of inclusion complexes.

#### 2.2.2. Powder X-ray Diffractometry (PXRD)

The PXRD profiles of the individual compositions, physical mixtures, and inclusion complexes are presented in Figure 2b. It shows that OL existed in amorphous powder with a diffraction peak at 2θ 20.86°, while beta-CD was typical crystal, exhibiting several characteristic crystalline peaks, such as 10°, 12.6°, 15.64°, 17.2°, 20.08°, 21.68°, 22.76°, 27.12°, and 34.96°. The results were coincided with what Mohandoss et al. reported [13]. The physical mixture of OL and beta-CD reserved the crystalline peaks of beta-CD, such as 10°, 12.48°, 22.8°, 27.04°, 34.72°, and so on, confirming the crystalline feature of the physical mixture. However, only a diffraction peak at 22.36° with attenuated intensity appears in the PXRD gram of beta-CD:OL, which implies that beta-CD:OL was in an amorphous state and might possess higher solubility. It provides evidence in supporting the formation of an inclusion complex between OL and beta-CD.

Figure 2b shows that HP-beta-CD has only one diffraction peak at 19°, demonstrating that HP-beta-CD belongs to amorphous powder, which is in accordance with the results reported by Mohandoss et al. [13]. The physical mixture of OL and HP-beta-CD presents a similar diffraction peak at 19.16° with approximate peak intensity. Though the HP-beta-CD:OL also displays one diffraction peak at 19.23°, the peak intensity is augmented with broadened width. The difference in PXRD profiles suggests that the inclusion complex may occur between OL and HP-beta-CD.

#### 2.2.3. Differential Scanning Calorimetry (DSC)

The DSC thermograms of the individual components, physical mixtures, and inclusion complexes are displayed in Figure 2c. No obvious peaks appeared in the thermogram of OL, owing to the small amount of OL used according to its proportion in the inclusion complex. Beta-CD had an endothermic peak around 117.21 °C, which aligns with the loss of water molecules. Furthermore, there are another two endothermic peaks at 315.22 °C and 323.09 °C which may originate from the melting and decomposition of beta-CD [14]. The physical mixture also showed the endothermic peak of water as well as the peak around melting point of beta-CD. However, compared to beta-CD, the intensity of the two peaks weakened substantially in the thermogram of the mixture. OL occupied the cavity of beta-CD and drove out some water molecules that stayed there before, leading to the dwindling of the peak corresponding to water loss. The existence of OL may also protect beta-CD from degrading and reduce the energy required in the decomposition reaction. As a result, the mixture produces a much smaller endothermic peak around 320 °C. The thermogram of beta-CD:OL tends to be a straight line with non-discernible endothermic peaks. The difference in DSC thermograms suggests the formation of inclusion complex beta-CD:OL.

HP-beta-CD had an endothermic peak around 100 °C and a complex endothermic peak from 300 to 363 °C, which corresponded with water dissociation from the compound and the melting point and decomposition of HP-beta-CD, respectively. The mixture showed a similar water endothermic peak and a broadened endothermic peak in line with HP-beta-CD. This complies with the rule that the mixture often has a wider melting range than the individual compounds. The inclusion complex also exhibited a water endothermic peak with a small shift to lower temperature, and a small and narrow endothermic peak around 350 °C. The DSC curves of free HP-beta-CD, physical mixture, and inclusion complex present apparent difference, verifying that the inclusion complex was formed between HP-beta-CD and OL.

### 2.3. Phase Solubility Diagrams

**Figure 3:**
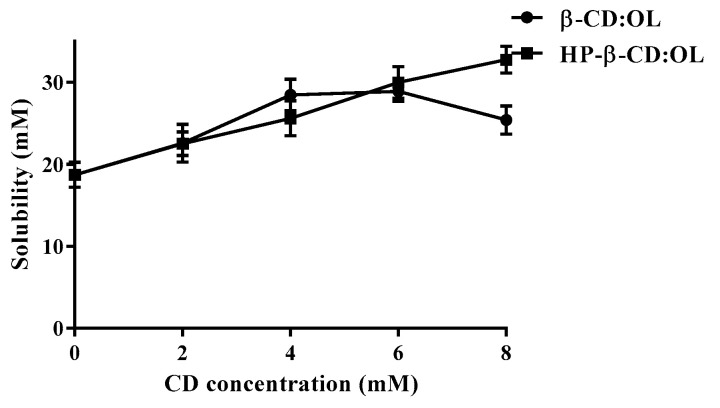
Phase solubility diagrams of OL with beta-CD and HP-beta-CD under 25 °C.

The solubility of OL in beta-CD and HP-beta-CD solutions at various concentrations is presented in Figure 3. The solubility diagram of OL with HP-beta-CD presents A (L) type. The OL content linearly increased with the CD concentration, suggesting a 1:1 stoichiometric complex between OL and HP-beta-CD [15]. The diagram of OL with beta-CD displays a curve which at first rises and then declines, indicating that a complex with a stoichiometry greater than 1 was formed. As the concentration of beta-CD increased, the turbidity of the solution rose as well, which may originate from the yield of more exposed free hydroxyls of beta-CD. As a result, the intermolecular action of beta-CD increased and the interplay between beta-CD and water weakened, leading to the decreased water solubility. Mourtzinos et al. prepared a beta-CD inclusion complex with olive leaf extract, which displayed an A (L) type diagram and the stoichiometry of 1:1 [16]. It manifests that OL and polyphenols in olive leaf extract were encapsulated into beta-CD in different inclusion fashions.

The solubility data of the two complexes fit well with Equation (1), depicting a complex form of 1:1 and 1:2 between the host molecule and the guest molecule [15]:

S_t_ − S_0_ = K_11_S_0_[CD] + K_11_K_12_S_0_[CD]^2^ (1)

The thermodynamic parameters of the complexing reaction, such as the changes of standard enthalpy (∆H^0^) and standard entropy (∆S^0^), were calculated according to the following van’s Hoff Equation (2) [17]:

(2) $$lnK=-\frac{∆H^{0}}{RT}+\frac{∆S^{0}}{R}$$

where *T* and *R* represent temperature and a gas constant, respectively. *K* is equal to K_11_.

The change of Gibbs free energy (∆G^0^) in the complexing was attained via Equation (3) [17]:

∆G^0^ = −*RT*ln*K* (3)

The calculated stability constants of beta-CD:OL and HP-beta-CD:OL are presented in Table 1. The correlation coefficients r^2^ under different temperatures were close to 1, indicating that Equation (1) well simulated the relationship between OL solubility and CD concentration, and the stability constants acquired from Equation (1) were reliable. The lg*K* of both OL inclusion complexes was around 2, implying an appropriate complexing. In general, the binding is considered unstable if lg*K* is beneath 2. On the other hand, the release of the guest molecule from the complex becomes difficult when lg*K* is over 4 [17]. The stability constants of most CD-based complexes decreased as temperature elevated [18,19]. However, the two OL inclusion complexes did not exhibit such trend. The K values fluctuated irregularly with temperature change. Heydari et al. also observed similar phenomena [20]. The possible explanation is that the formation of OL complexes needs energy supply. However, high temperature leads to the hydrogen bond breaking between OL and CD accompanied with the decreased stability. Sufficient energy supply along with less dissociation of intermolecular hydrogen bonds will yield high stability constants of OL inclusion complexes. Under various temperatures, the K of beta-CD:OL was generally higher than that of HP-beta-CD:OL, demonstrating that beta-CD:OL is more stable over HP-beta-CD:OL.

The thermodynamic parameters for beta-CD:OL and HP-beta-CD:OL are displayed in Table 2. The **∆G^0^** of both complexes were negative, manifesting that the complexing reaction was spontaneous [21,22]. Meanwhile, the reaction was endothermic (∆H^0^ >0), implying that energy was needed to destroy the intramolecular hydrogen bonds of CD and OL. It also indicates that the released energy from the formation of hydrogen bonds and vander Waals forces between CD and OL was lower than the energy required to break the intramolecular hydrogen bonds. Compared to beta-CD:OL, HP-beta-CD:OL had much greater ∆H^0^, implying that OL generated weaker interaction with HP-beta-CD. The change of entropy is associated with the desolvation process [20]. The positive ∆S^0^ means entropy is the principle driving force for the formation of OL inclusion complexes.

### 2.4. Stability Test

OL content on day 0 was set as 100%, respectively. The contents determined on day 5 and 10 were compared with the data of day 0. The results are shown in Table 3. OL was sensitive to light, temperature and humidity. It was extremely unstable when exposed to high humidity. The degrading amount reached 34% after OL was kept in relative humidity (R.H.) of 92.5% for 10 d. Compared to the free component, both inclusion complexes obviously increased the stability of OL (*p* < 0.05). Moreover, beta-CD:OL exhibited more stable feature than HP-beta-CD:OL in various extreme conditions, especially under high humidity. Placed in R.H. of 92.5% for 10 d, nearly 30% OL degraded in HP-beta-CD complex, whereas beta-CD complex preserved 94.68% of OL (Table 3). CD are capable of protecting the components from degrading [23]. Li et al. reported that the stability of cryptotanshinone inclusion complex with beta-CD was lower than that of the complex with HP-beta-CD [24]. In another study, the beta-CD inclusion complex of oxaliplatin presented higher stability over the HP-beta-CD complex [25]. The interaction between host and guest molecules may be a dominant factor to the stability of a complex. Polar compounds may construct more stable complexes with beta-CD due to plentiful hydrogen bond linkages. Likewise, HP-beta-CD complexes with lipophilic compounds will be more stable owing to more interplay between HP-beta-CD and the compound via van der Waals forces.

### 2.5. Molecular Docking and IR

The binding energy of beta-CD: OL and HP-beta-CD: OL was −6.1 and −24.66 KJ/mol, respectively, indicating that OL had a stronger affinity with HP-beta-CD. As shown in Table 4, both inclusion complexes had several intermolecular conventional hydrogen bonds and carbon hydrogen bonds. Here the carbon hydrogen bond was defined as C-H...O and C-O...H, with a more remote distance than with the conventional hydrogen bond.

**Figure 4:**
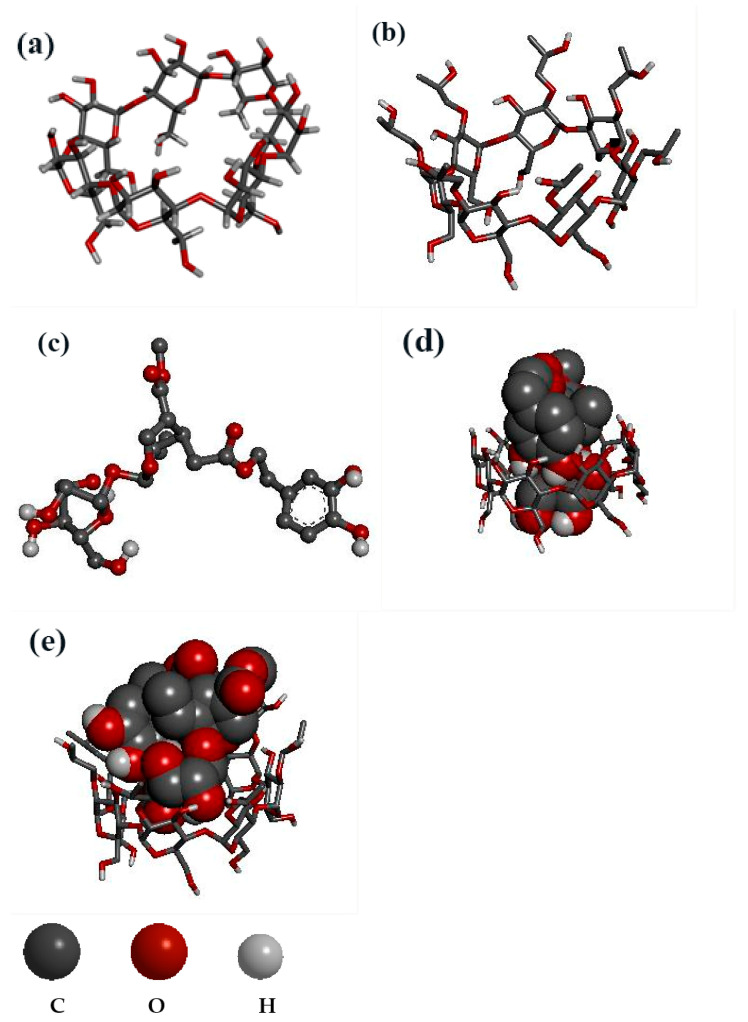
Molecular docking studies with ball-and-stick and sphere (CPK) representations: (**a**)beta-CD, (**b**) HP-beta-CD, (**c**) OL, (**d**) beta-CD:OL, (**e**) HP-beta-CD:OL. The abbreviations are the same as in Figure 1.

As presented in Figure 4, both beta-CD and HP-beta-CD have a large lumen (Figure 4a,b), and OL (Figure 4c) entered the CD to form inclusion complexes (Figure 4d,e). OL has two ester bonds that are sensitive to humidity. Its two phenol hydroxyls are easily oxidized upon exposure to light and high temperature. The two phenolic hydroxyls of OL entered the cavity of beta-CD (Figure 4d) and formed two conventional hydrogen bonds. In addition, one of the ester bonds of OL yielded another conventional hydrogen bond with beta-CD (Table 4). In contrast, the two phenolic hydroxyls of OL failed to completely penetrate into the cavity of HP-beta-CD (Figure 4e), and only one of the phenolic hydroxyls generated a hydrogen bond with HP-beta-CD (Table 4). In addition, the two ester bonds of OL were partly exposed outside the cavity of HP-beta-CD (Figure 4e), which was also confirmed by the IR spectrum. The peak intensity of carbonyl around 1700 cm^−1^ was OL > HP-beta-CD:OL > beta-CD:OL (Figure 2a), showing that, to some degree, both inclusion complexes shielded ester bonds of OL via the hydrogen bond between carbonyl and CD. Nevertheless, with respect to beta-CD:OL, HP-beta-CD:OL exposed more ester bonds outside and increased the risk of being hydrolyzed under high humidity. Carbon hydrogen bonds of HP-beta-CD:OL were major binding forces between glucose hydroxyls of HP-beta-CD and OL (Table 4). The results from docking and IR infers that owing to the stronger binding affinity between HP-beta-CD and OL, HP-beta-CD:OL could be constructed more easily. However, the phenol hydroxyls and ester bonds of OL were not well protected by HP-beta-CD, leading to the decreased stability of HP-beta-CD:OL. The results, which were consistent with the thermodynamic parameters, well explained why beta-CD:OL was more stable than HP-beta-CD:OL.

### 2.6. Reactive Phenol Hydroxyls

**Figure 5:**
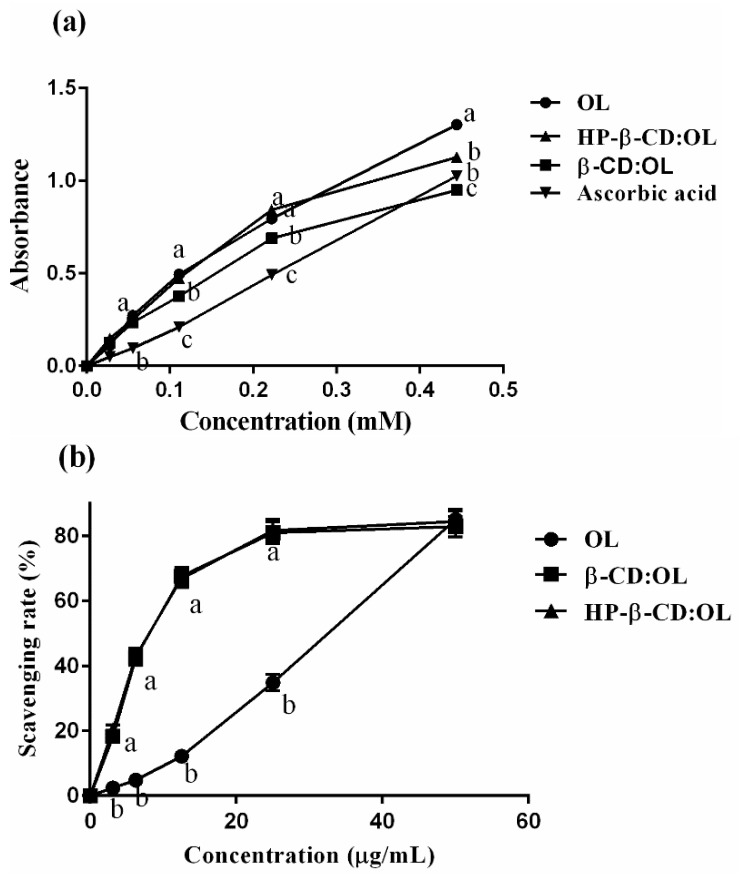

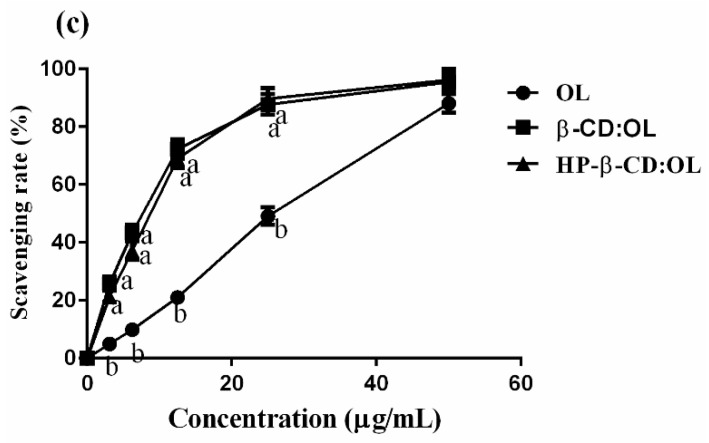
Comparison of OL, HP-beta-CD:OL and beta-CD:OL: the number of reactive phenol hydroxyls (**a**), scavenging rates against DPPH (**b**) and ABTS^+^ radicals (**c**). The reactive phenol hydroxyls were estimated via the ferric reducing power of the samples. The letters a, b, and c represent significant differences (*p* < 0.05 in (**a**), *p* < 0.01 in **b**,**c**).

The process of absorbance varying with the concentration is displayed in Figure 5a. Phenols of OL have the capacity of reducing Fe^3+^ into Fe^2+^, which exists in the form of K_4_Fe(CN)_6_. Subsequently, the reduced product combines with ferric chloride and yields Prussian blue, which is soluble in glacial acetic acid with a maximum absorbance at 700 nm. Figure 5a shows that though the absorbance of both samples and ascorbic acid increases in a concentration dependent mode, free ascorbic acid and OL present a linear ascending trend, while turning points appeared in the curves of inclusion complexes. When concentration reached the turning points, the absorbance began to increase slowly. OL exhibited strong reducing power, even significantly higher than that of ascorbic acid at the concentration below 0.3 mM (*p* < 0.05). Since the reducing ability of OL originates from its two reactive phenol hydroxyls in the structure, the absorbance is positively related with phenol numbers. Among the three samples, beta-CD:OL had the lowest absorbance in the range of 0.1 to 0.45 mM, demonstrating that minimum reactive phenol hydroxyls were present in beta-CD:OL. The absorbance of HP-beta-CD:OL and OL were similar to each other at low concentrations. However, the reducing power of OL quickly exceeded that of HP-beta-CD:OL when the concentration amounted to 0.45 mM (*p* < 0.05). The study indicates that both inclusion complexes shielded the phenol hydroxyls of OL. Beta-CD:OL displayed more potent capacity in protecting reactive phenol hydroxyls, thus diminishing the yield of Prussian blue. In addition, phenols are the major source of the bitter taste [26]; the decrease of reactive phenols means less bitter taste will be present.

### 2.7. Antioxidant Activities

The capacity of OL and its two inclusion complexes in scavenging free radicals is shown in Figure 5b,c. The inclusion complexes displayed significantly stronger power than free OL in quenching DPPH and ABTS^+^ radicals when OL concentration was below 40 µg/mL (*p* < 0.01). The CL_50_ and calculated antioxidant activity expressed as g Trolox equivalents (g TE)/g OL are displayed in Table 5. The two inclusion complexes have equivalent capacity against DPPH radicals, which was 5 folds that of free OL. The strength of beta-CD:OL and HP-beta-CD:OL to scavenge ABTS^+^ radicals was 2.87 and 2.32 folds that of OL, respectively. The powerful antioxidant activities of the inclusion complexes may stem from the higher solubility as well as better compatibility with polar free radicals [27], which assists the entrapment and clearance of the radicals.

DPPH method is based on hydrogen atom transfer [28]. Antioxidants need to provide hydrogen atoms to bind and eliminate DPPH radicals. In contrast, the ABTS^+^ method is on the ground of electron transfer [29]. The clearance of ABTS^+^ radicals depends on the electrons liberated by antioxidants. Compared to OL, the inclusion complexes can produce more hydrogen atoms as well as electrons to quench both DPPH and ABTS^+^ radicals and present stronger antioxidant activity. Intermolecular hydrogen bonds of inclusion complexes also contribute to the enhanced strength [30].

Free OL exhibited a linear increase of activity with the concentration. The scavenging rates of the two inclusion complexes also presented a concentration-dependent mode when OL concentration was below 25 µg/mL. The strength reached the peak level around 80% at 25 µg/mL. Afterward, the quenching rates maintained 80% without obvious increase, even when the concentration amounted to 50 µg/mL. The possible explanation was that the release of OL from the inclusion complexes became difficult at high concentrations [31].

Irakli et al. used ultrasound to extract OL and other phenolic compounds from olive leaves [32]. The DPPH scavenging capacity of the extract was around 40 mg TE/g, which was only 27% of the strength of OL measured by us. Wang et al. also observed higher antioxidant activity of OL compared to the phenol extract [33]. In most cases, CD inclusion complexes were capable of enhancing the antioxidant activity of the free compound [34,35]. Occasionally, the radical-quenching capacity of the compound was decreased after being encapsulated by CD [36]. The possible reason was that the functional groups responsible for snatching free radicals were shielded by CD.

### 2.8. Solubility Test

The water solubility of OL, beta-CD:OL and HP-beta-CD:OL, which was determined based on pure OL, was 12.78 ± 0.36, 75.82 ± 1.27, and 77.52 ± 1.41 mg/mL, respectively. The solubility of OL was increased fivefold after it was formulated into inclusion complexes. Since the substitute of hydroxypropyl destroyed the intermolecular action among HP-beta-CD, the solubility of the HP-beta-CD complex is usually higher than that of the beta-CD complex [37,38]. However, in our study, there was no evident solubility difference for the two OL inclusion complexes. The possible explanation was that OL combined with beta-CD via hydrogen bond linkage, decreasing the exposed free hydroxyls and successive intermolecular action among beta-CD molecules. As a result, the interaction between beta-CD and water was enhanced, leading to the augmented solubility of the beta-CD complex.

### 2.9. Permeation through Mouse Small Intestine

**Figure 6:**
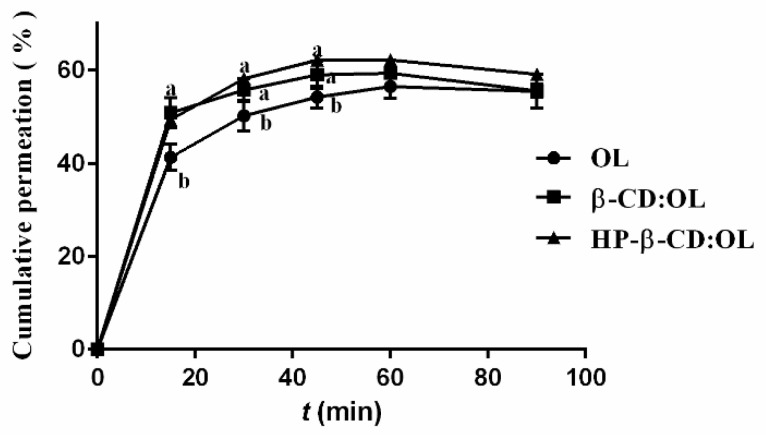
The Permeation curves of OL and its inclusion complexes across mouse small intestine. Different letters represent significant differences (*p* < 0.05).

The permeation curves of OL and its inclusion complexes across mouse small intestine are shown in Figure 6. It seems that due to the polarity of OL, its intestinal absorption was poor. Even in the form of inclusion complex, only less than 60% OL passed through the intestine. During the first 45 min, the absorption of the two inclusion complexes was significantly higher than that of free OL (*p* < 0.05). After 1 h, the cumulative absorption amounts showed a slight decrease due to the presence of intestinal enzymes. In practice, researchers prefer using HP-beta-CD rather than beta-CD to enhance the bioavailability of insoluble drugs due to the stronger capacity of HP-beta-CD in increasing the water solubility of a compound [39,40]. Moreover, HP-beta-CD was reported to have an inhibitory effect on P-gP ATPase and restrain the efflux of P-gP, while beta-CD did not show any inhibitory activity [41]. In our study, although HP-beta-CD complex of OL permeated through the small intestine a little faster than the beta-CD complex, there was no statistical difference (*p* > 0.05). The interaction between OL and hydroxyl groups of beta-CD via hydrogen bonds reduced the polarity of beta-CD, which enhanced both the solubility and intestinal permeability of the beta-CD complex. For this reason, the intestinal absorption of beta-CD:OL was comparable to HP-beta-CD:OL.

The permeation process of free OL and inclusion complexes was simulated by different equations. The transport profile of free OL fitted the mathematical pattern of logarithm normal distribution. The equation was *Y* = 11.67 + 25.55lg*t* (*r*^2^ = 0.9908). The intestinal permeation process of beta-CD:OL conformed to the Weibull model with the equation of lnln [1/(*Y*_∞_ − *Y*)] = −0.1275 + 0.2952ln*t* (*r*^2^ = 0.9783). The transmembrane of HP- beta-CD:OL also followed the Weibull model with the equation of lnln[1/(*Y*_∞_ − *Y*)] = −0.7403 + 0.5078ln*t* (*r*^2^ = 0.9664).

## 3. Materials and Methods

### 3.1. Materials

Beta-CD and HP-beta-CD were purchased from Shanghai Aladdin Biochemical Technology Corp. (Shanghai, China). OL, with a purity over 90%, was kindly supplied by Lanzhou Institute of Chemical Physics, Chinese Academy of Science (Lanzhou, China). 2,2-Diphenyl-1-picrylhydrazyl (DPPH) was obtained from Tokyo Chemical Industry Company (Tokyo, Japan). 2,2′-Azino-bis(3-ethylbenzothiazoline)-6-sulfonate (ABTS^+^) was provided by the Nanjing Oddfoni Biological Technology Co., Ltd. (Nanjing, China). All other chemicals were of analytical grade and were obtained from Chengdu Kelon Chemicals Corp. (Chengdu, China).

### 3.2. Preparation of Inclusion Complexes

Beta-CD of 10 g was dissolved in 10 mL distilled water at 50 °C. An OL of 4 g was dissolved in 50% ethanol of 1mL. They were mixed together and stirred constantly by an agitator (S10-3, Shanghai Si Le Instrument Corp., Shanghai, China) at 50 °C for 3 h. Afterward, the solvent was removed by rotary thin film evaporation (B-260, Shanghai Yarong Biochemical Instrument Factory, Shanghai, China). The remnant was vacuum dried to constant weight and kept at room temperature (22–25 °C). The inclusion complex of HP-beta-CD was prepared following the above procedures, except that the mass ratio of the material to OL was 3:1.

The complex of 10 mg was dispersed in a volumetric flask of 25 mL with distilled water, from which 2 mL was drawn out and centrifugated at 20,000× *g* under 4 °C for 10 min. The supernatant was analyzed by HPLC (LC-20AD, Shimadzu, Japan) after quantitative dilution. The samples were isolated on a Cosmosil C18 column (4.6 mm × 150 mm, 5 µm). The detection wavelength was set at 279 nm with the column temperature of 35 °C. The mobile phase consisted of acetonitrile-water (22:78) with the flow rate of 1.0 mL/min. The injection volume was 20 µL. The encapsulation efficiency (*EE*) and drug loading (*DL*) was calculated by the following equations:

*EE* = (*W_t_* − *W_s_)*/*W_t_* × 100% (4)

*DL* = (*W_t_* − *W_s_*)/*W_i_* (5)

where *W_t_* represents the total amount of OL (mg). *W_s_* represented OL content in the supernatant (mg). *W_i_* was the total amount of inclusion complex (mg).

### 3.3. Confirming the Formation of Inclusion Complex

#### 3.3.1. FT-IR Analysis

Samples of OL, beta-CD, HP-beta-CD, physical mixtures of the host molecule and guest molecule, beta-CD:OL and HP-beta-CD:OL were prepared according to the complex formula. FT-IR spectra were scanned in the range of 400–4000 cm^−1^ [11], respectively, by an FT-IR spectrometer (Spectrum Two Infrared Spectrometer, Perkin Elmer, Shanghai, China). Before the scan, the samples were compressed into thin tablets with KBr, respectively.

#### 3.3.2. PXRD Analysis

The PXRD patterns of the individual compounds, physical mixtures, and inclusion complexes were recorded by an X-ray diffractometer (DX-2700B, Dandong Haoyuan Instrument Corp., Dandong, China) using Cu-kα radiation, with the voltage of 40 kV and current of 30 mA. All samples were scanned in the 2θ range of 10–80° at a scan rate of 5°/min and a wavelength of 1.5405 Å [13].

#### 3.3.3. DSC Analysis

DSC analysis was carried out on a DSC-60 cell differential scanning calorimeter (DSC-60A, Shimadzu, Japan). The samples were weighed and put on an aluminum pan, respectively, according to its proportions in the formula. Another pan without sample was set as the reference. The pans of the sample and reference were heated together from 20 °C to 400 °C at a rate of 10 °C/min in a dynamic high purity nitrogen flow of 50 mL/min.

### 3.4. Phase Solubility Test

The tubes and distilled water were autoclaved before the experiment was conducted. Then, a series of beta-CD and HP-beta-CD solutions from 0 to 8 mM were prepared with water, respectively. OL was added to the solutions under ultrasound treatment until the obvious sediment was observed. Subsequently, the solutions were shaken under 25, 35, 40 and 45 °C for 72 h, respectively [17], followed by being centrifuged at 20,000× *g* under 4 °C for 10 min. The supernatants were diluted with water and the contents of OL were determined by HPLC, as described above.

### 3.5. Stability Test

OL, inclusion complexes of beta-CD:OL and HP-beta-CD:OL, were exposed to light (5000 lux, RXZ-300B, Ningbo Southeast Instrument Corp., Ningbo, China) at room temperature, 60 °C, R.H. of 75% and 92.5%, respectively, to examine the effect of light, temperature, and humidity on the stability of OL and its inclusion complexes. The experiments were conducted for 10 d. The content of the samples was determined by HPLC on day 0, 5, and 10, respectively.

### 3.6. Molecular Docking

To further scrutinize the formation mechanisms of the inclusion complexes beta-CD:OL and HP-beta-CD:OL, theoretical calculations were performed using the most probable structures determined by Autodock. The crystal structure of beta-CD was obtained from the RCSB Protein Data Bank (PDB ID: 4YEF) [42] and was modified to obtain HP-beta-CD by Gaussian View 6.0. Its conformation was then optimized by the PM3 method. Molecular docking between CD and OL was conducted by Autodock 4.2 software. OL was connected to the cavities of beta-CD and HP-beta-CD, respectively, and Discovery Studio 2020 software was used to visually analyze the docking results. The docking with low binding energy and strong affinity was considered as stable conformation.

### 3.7. Reactive Phenol Hydroxyls

OL, beta-CD:OL and HP-beta-CD:OL solutions were prepared with water, in which OL concentrations ranged from 0 to 0.44 mM. The sample of 1 mL was mixed with 2.5 mL of 0.2 M phosphate buffer solution (PBS, pH 6.8) and 2.5 mL of 1% K_3_Fe(CN)_6_ (*w*/*v*), incubated under 50 °C for 20 min, and cooled to ambient temperature. Subsequently, 2.5 mL of 10% trichloroacetic acid (*w*/*v*) was added, from which 2.5 mL was withdrawn, mixed with 2.5 mL of distilled water and 0.5 mL of 0.1% ferric chloride (*w*/*v*), and reacted under ambient temperature for 10 min. Afterward, 1.5 mL of glacial acetic acid was added to solubilize the product and the absorbance at 700 nm was determined. Meanwhile, the background absorbance was measured by using distilled water in place of K_3_Fe(CN)_6_ and ferric chloride. Ascorbic acid in the scope of 0 to 0.44 mM was set as the control.

### 3.8. Antioxidant Activity

#### 3.8.1. Scavenging DPPH Radicals

The activity of OL, beta-CD:OL and HP-beta-CD:OL to scavenge DPPH radicals was assessed according to the methods proposed by Jing et al. [43]. Briefly, OL, beta-CD:OL and HP-beta-CD:OL were prepared into a series of solutions in which OL concentrations ranged from 5 to 50 µg/mL. The sample of 1 mL was then mixed with 2 mL DPPH of 0.02 mg/mL, stood in the dark for 30 min, and the absorbance at 517 nm was determined (*A*). In addition, 1 mL distilled water was in place of the sample and manipulated as mentioned above. The absorbance at 517 nm was marked as *A*_0_. The background response was also determined by mingling 1 mL sample with 2 mL ethanol (*A_b_*). The scavenging rate was calculated according to the Equation (6). Trolox was the positive control. The clearance curve was plotted using a scavenging rate versus OL concentration. The concentration to clear 50% free radicals (CL_50_) was calculated via the curve and expressed as g TE/g OL.

Scavenging rate = (*A*_0_ − (*A* − *A_b_*))/*A*_0_ ∗100% (6)

#### 3.8.2. Scavenging ABTS^+^ Radicals 

The measurement was conducted as proposed by Yao et al. [44]. In general, ABTS^+^ solution was prepared by mixing 7 mM ABTS^+^ with 2.45 mM potassium persulfate (1:1, *v*/*v*), and reacting in the dark for 12 to 16 h. The concentration of ABTS^+^ was then adjusted with water to make *A_0_* in the range of 0.68 to 0.72. The sample of 1 mL was blended with ABTS^+^ of 2 mL, stood in the dark for 6 min, and the absorbance at 734 nm was measured (*A*). Furthermore, *A_0_* and *A_b_* were determined as described above. The scavenging rate was calculated according to Equation (6). CL_50_ was obtained via the clearance profile.

### 3.9. Water Solubility

OL and its inclusion complexes were continuously added to 1 mL of water, respectively, until obvious precipitation was observed. The suspensions were shaken at 50 rpm under 25 °C for 24 h, followed by centrifugation at 20,000× *g* under 4 °C for 10 min. The supernatant was diluted quantitatively with water and determined by HPLC, as described above.

### 3.10. Permeating Test Using Mouse Small Intestine

Male Kunming mice, weighing 20–22 g, were purchased from Chengdu Dashuo Laboratory Animal Corp. (Chengdu, China). The mouse experiments complied with the guidance with regard to Animal Care and Use of Chengdu University and were approved by the ethics committee of the university. The mice were fasted but free to drink water for 12 h prior to the experiment. Afterward, the mice were sacrificed by breathing carbon dioxide. The small intestine along with the stomach was isolated from the body quickly. The content in small intestine was rinsed out by infusing physiological saline via the stomach inlet. The treated small intestine was immersed in saline at 37 °C for 1 h, cut into 10 cm from the stomach inlet, and loaded with a 1 mL sample, respectively [45]. OL concentration in the samples was 1 mg/mL.

After the small intestine was filled with the sample, it was transferred to a 50 mL conical flask, which contained 20 mL of PBS (pH 7.4), and then was pre-incubated in a 37 °C water bath shaker (SHZ-B water bath thermostatic oscillator, Shanghai Boxun Industrial Corp., Shanghai, China). The shaker was started at the speed of 50 rpm. At designated time intervals, 0.5 mL release medium was withdrawn for HPLC analysis and 0.5 mL fresh medium incubated in 37 °C was replenished into the flask. The assay condition of HPLC was as described above. The response of the OL solution of 50 µg/mL was set as the reference. The cumulative transmembrane rates of samples were obtained by comparing peak areas with the reference. The in vitro permeation curves were plotted using cumulative transmembrane rate versus time. The absorption process was simulated by the mathematical patterns of monoexponential, logarithm normal distribution, Higuichi, and Weibull, as expressed by the following Equations (7) to (10), respectively.

lg(*Y_∞_* − *Y*) = a + b*t* (7)

*Y* = a + blg*t* (8)

(9) $$Y=a+b\sqrt{t}\;$$

lnln[1⁄((*Y_∞_* − *Y*))] = a + bln*t* (10)

where *Y_∞_* and *Y* represents the cumulative permeation rate at the equilibrium state and *t* min, respectively. The letters a and b were the coefficients of the equations.

### 3.11. Statistical Analysis

Data were presented as mean ± SD from triple experiments. The statistical analysis was conducted by Excel 2016 (Microsoft Corporation, Redmond, DC, USA). The difference was considered significant when *p* ≤ 0.05, which was determined by one-way variance of analysis (ANOVA) and a Tukey’s test.

## 4. Conclusions

Beta-CD bound with OL according to the stoichiometry of 1:1 and 1:2, while HP-beta-CD linked with OL were based on the stoichiometry of 1:1. The calculated lg*K* (25 °C) and binding energy of beta-CD:OL and HP-beta-CD was 2.32 versus1.98, and −6.1 versus −24.66 KJ/mol, respectively. Beta-CD:OL displayed stronger capacity than HP-beta-CD:OL in preventing OL from degrading upon exposure to high humidity, high temperature, and light, which was consistent with the stability constants, but contrary to the binding energy. Molecular docking, peak intensity of carbonyl in IR spectra, and ferric reducing power indicated that the exposure of either ester bonds or phenol hydroxyls of the samples was free OL > HP-beta-CD:OL > beta-CD:OL, verifying that beta-CD shielded the unstable groups of OL more efficiently than HP-beta-CD. The reduced reactive phenol hydroxyls also implies the bitter taste will be diminished. In addition, the inclusion complexes significantly increased the antioxidant activity, solubility and intestinal permeation of OL (*p* < 0.05). With respect to beta-CD:OL, HP-beta-CD:OL had higher solubility and intestinal absorption, but with no statistical difference. The study demonstrates that the lower binding energy of a complex does not always correspond to higher stability. Though HP-beta-CD is a more popular material used to prepare inclusion complexes, beta-CD may be more efficient in protecting a compound with multiple hydroxyls, whereas the intestinal absorption is comparable to the HP-beta-CD complex.

## Figures and Tables

**Table 1:** The calculated stability constants of beta-CD:OL and HP-beta-CD:OL under different temperatures.

Temperature (K)	Beta-CD:OL		HP-beta-CD:OL	
	lg*K*	*r* ^2^	lg*K*	*r* ^2^
298	2.316	0.9669	1.978	0.9925
308	2.327	0.9534	2.167	0.9657
313	1.910	0.9871	1.758	0.8662
318	2.118	0.9999	2.141	0.9992

**Table 2:** Thermodynamic parameters for beta-CD:OL and HP-beta-CD:OL.

Sample	∆G^0^ (25 °C, kJ mol^−1^)	∆H^0^ (kJ mol^−1^)	∆S^0^ (J mol^−1^ K^−1^)
Beta-CD:OL	−13.21	1.96	50.91
HP-beta-CD:OL	−11.29	33.08	148.90

**Table 3:** The effects of light, temperature, and humidity on the stability of OL and its inclusion complexes.

Condition	Time (d)	OL (%)	Beta-CD:OL (%)	HP-Beta-CD:OL (%)
Light (25 °C)	0	100.00 ± 0.50	100.00 ± 0.61	100.00 ± 0.44
	5	94.99 ± 0.61	99.10 ± 0.47	93.96 ± 0.54
	10	93.18 ± 0.52	98.67 ± 0.34	93.78 ± 0.52
Temperature (60 °C)	0	100.00 ± 0.55	100.00 ± 0.56	100.00 ± 0.64
	5	96.46 ± 0.74	98.30 ± 0.35	98.69 ± 0.65
	10	87.37 ± 0.38	97.98 ± 0.54 *	95.83 ± 0.56 *
Humidity (R.H. 75%)	0	100.00 ± 0.34	100.00 ± 0.62	100.00 ± 0.35
	5	76.69 ± 0.54	96.44 ± 0.55 **	90.90 ± 0.46 **
	10	74.91 ± 0.39	92.16 ± 0.44 **	87.87 ± 0.56 *
Humidity (R.H. 92.5%)	0	100.00 ± 0.35	100.00 ± 0.38	100.00 ± 0.44
	5	75.42 ± 0.56	94.95 ± 0.34 **	79.84 ± 0.48
	10	65.98 ± 0.54	94.68 ± 0.46 **	70.88 ± 0.54

OL: oleuropein; beta-CD:OL: OL inclusion complex with beta-CD; HP-beta-CD:OL: OL inclusion complex with HP-beta-CD. *, **: *p* < 0.05, *p* < 0.01, compared to free OL.

**Table 4:** Nonbonding force and distance between OL and beta-CD and HP-beta-CD molecules.

Inclusion Material	Type	Distance(Å)
beta-CD	Conventional Hydrogen Bond	2.179
	Conventional Hydrogen Bond	2.390
	Conventional Hydrogen Bond	2.500
	Carbon Hydrogen Bond	3.665
	Carbon Hydrogen Bond	3.311
	Carbon Hydrogen Bond	3.381
	Carbon Hydrogen Bond	3.693
	Carbon Hydrogen Bond	3.287
HP-beta-CD	Conventional Hydrogen Bond	1.835
	Conventional Hydrogen Bond	2.777
	Carbon Hydrogen Bond	3.653
	Carbon Hydrogen Bond	3.255
	Carbon Hydrogen Bond	3.543
	Hydrophobic	4.669
	Hydrophobic	4.673
	Hydrophobic	5.413

**Table 5:** CL_50_ of OL, beta-CD:OL and HP-beta-CD:OL against DPPH and ABTS^+^.

Sample	CL_50_ (µg/mL)		g TE/g OL	
	DPPH	ABTS^+^	DPPH	ABTS^+^
OL	40.10	26.17	0.1487	0.1315
Beta-CD:OL	9.96	9.12	0.5993	0.3769
HP-beta-CD:OL	9.87	11.27	0.6045	0.3052
Trolox	5.97	3.44	1.0000	1.0000

The calculation was based on OL. CL_50_: OL concentration to clear 50% free radicals. TE: Trolox equivalent. Other abbreviations are as Table 1.

## Data Availability

Data are included within the article.

## References

[1] Santini S.J., Porcu C., Tarantino G., Balsano C. (2020). Antioxidant and anti-inflammatory effect of oleuropein in hepatic steatosis. Digest. Liver Dis..

[2] Al-Quraishy S., Othman M.S., Dkhil M.A., Abdel Moneim A.E. (2017). Olive (Olea europaea) leaf methanolic extract prevents HCl/ethanol-induced gastritis in rats by attenuating inflammation and augmenting antioxidant enzyme activities. Biomed. Pharmacother..

[3] Alarcón de la Lastra C., Barranco M.D., Motilva V., Herrerías J.M. (2001). Mediterranean diet and health: Biological importance of olive oil. Curr. Pharm. Des..

[4] Tuck K.L., Hayball P.J. (2002). Major phenolic compounds in olive oil: Metabolism and health effects. J. Nutr. Biochem..

[5] Al-Azzawie H.F., Alhamdani M.S. (2006). Hypoglycemic and antioxidant effect of oleuropein in alloxan-diabetic rabbits. Life Sci..

[6] Jacob S., Nair A.B. (2018). Cyclodextrin complexes: Perspective from drug delivery and formulation. Drug Dev. Res..

[7] Yu B., Wang J.P., Zhang H.X., Jin Z.Y. (2011). Investigation of the interactions between the hydrophobic cavities of cyclodextrins and pullulanase. Molecules.

[8] Amasya G., Bakar-Ates F., Wintgens V., Amiel C. (2020). Layer by layer assembly of core-corona structured solid lipid nanoparticles with beta-cyclodextrin polymers. Int. J. Pharm..

[9] Guo Z., Wu F., Singh V., Guo T., Ren X., Yin X., Shao Q., York P., Patterson L.H., Zhang J. (2017). Host-guest kinetic interactions between HP-beta-cyclodextrin and drugs for prediction of bitter taste masking. J. Pharm. Biomed. Anal..

[10] Pacheco P.A., Rodrigues L.N.C., Ferreira J.F.S., Gomes A.C.P., Verissimo C.J., Louvandini H., Costa R.L.D., Katiki L.M. (2018). Inclusion complex and nanoclusters of cyclodextrin to increase the solubility and efficacy of albendazole. Parasitol. Res..

[11] Srinivasan K., Stalin T. (2014). Study of inclusion complex between 2,6-dinitrobenzoic acid and beta-cyclodextrin by 1H NMR, 2D 1H NMR (ROESY), FT-IR, XRD, SEM and photophysical methods. Spectrochim. Acta Part A.

[12] Barman B.K., Rajbanshi B., Yasmin A., Roy M.N. (2018). Exploring inclusion complexes of ionic liquids with alpha- and beta-cyclodextrin by NMR, IR, mass, density, viscosity, surface tension and conductance study. J. Mol. Struct..

[13] Mohandoss S., Atchudan R., Immanuel Edison T., Mandal T.K., Palanisamy S., You S., Napoleon A.A., Shim J.J., Lee Y.R. (2019). Enhanced solubility of guanosine by inclusion complexes with cyclodextrin derivatives: Preparation, characterization, and evaluation. Carbohydr. Polym..

[14] Aigner Z., Berkesi O., Farkas G., Szabó-Révész P. (2012). DSC, X-ray and FTIR studies of a gemfibrozil/dimethyl-beta-cyclodextrin inclusion complex produced by co-grinding. J. Pharm. Biomed. Anal..

[15] Uzqueda M., Martίn C., Zornoza A., Sάnchez M., Vélaz I. (2010). Physicochemical characterization of terbinafine-cyclodextrin complexes in solution and in the solid state. J. Incl. Phenom. Macrocycl. Chem..

[16] Mourtzinos I., Salta F., Yannakopoulou K., Chiou A., Karathanos V.T. (2007). Encapsulation of olive leaf extract in beta-cyclodextrin. J. Agric. Food Chem..

[17] Ol’khovich M.V., Sharapova A.V., Perlovich G.L., Skachilova S.Y., Zheltukhin N.K. (2017). Inclusion complex of antiasthmatic compound with 2-hydroxypropyl-beta-cyclodextrin: Preparation and physicochemical properties. J. Mol. Liq..

[18] Ol’khovich M.V., Sharapova A.V., Lavrenov S.N., Blokhina S.V., Perlovich G.L. (2015). Inclusion complexes of hydroxypropyl-beta-cyclodextrin with novel cytotoxic compounds: Solubility and thermodynamic properties. Fluid Phase Equilibr..

[19] Saroj M.K., Payal R., Jain S.K., Sharma N., Rastogi R.C. (2018). Investigation of indole chalcones encapsulation in beta-cyclodextrin: Determination of stoichiometry, binding constants and thermodynamic parameters. J. Incl. Phenom. Macro..

[20] Heydari S., Kakhki R.M. (2017). Thermodynamic study of complex formation of beta-cyclodextrin with ibuprofen by conductometric method and determination of ibuprofen in pharmaceutical drugs. Arab. J. Chem..

[21] Usacheva T.R., Volynkin V.A., Panyushkin V.T., Lindt D.A., Pham T.L., Nguyen T.T.H., Le T.M.H., Alister D.A., Kabirov D.N., Kuranova N.N. (2021). Complexation of cyclodextrins with benzoic acid in water-organic solvents: A solvation-thermodynamic approach. Molecules.

[22] Usacheva T., Kabirov D., Beregova D., Gamov G., Sharnin V., Biondi M., Mayol L., D’Aria F., Giancola C. (2019). Thermodynamics of complex formation between hydroxypropyl-beta-cyclodextrin and quercetin in water-ethanol solvents at T = 298.15 K. J. Therm. Anal. Calorim..

[23] Do Carmo C.S., Pais R., Simplicio A.L., Mateus M., Duarte C.M.M. (2017). Improvement of aroma and shelf-life of non-alcoholic beverages through cyclodextrins-limonene inclusion complexes. Food Bioprocess Technol..

[24] Li J.F., Wei Y.X., Ding L.H., Dong C. (2003). Study on the inclusion complexes of cryptotanshinone with beta-cyclodextrin and hydroxypropyl-beta-cyclodextrin. Spectrochim. Acta A.

[25] Zhang D., Zhang J.Q., Jiang K.M., Li K., Cong Y.W., Pu S.P., Jin Y., Lin J. (2016). Preparation, characterisation and antitumour activity of beta-, gamma- and HP-beta-cyclodextrin inclusion complexes of oxaliplatin. Spectrochim. Acta Part A.

[26] Mateos R., García-Ortíz Civantos C., Castro J., Garcia-Mesa J.A. (2005). Direct spectrophotometric determination of bitterness in virgin olive oil without prior isolation by pH gradient. J. Agric. Food Chem..

[27] Jullian C., Orosteguis T., Pérez-Cruz F., Sánchez P., Mendizabal F., Olea-Azar C. (2008). Complexation of morin with three kinds of cyclodextrin: A thermodynamic and reactivity study. Spectrochim. Acta Part A.

[28] Ge X., Jing L., Zhao K., Su C., Zhang B., Zhang Q., Han L., Yu X., Li W. (2021). The phenolic compounds profile, quantitative analysis and antioxidant activity of four naked barley grains with different color. Food Chem..

[29] Thaipong K., Boonprakob U., Crosby K., Cisneros-Zevallos L., Hawkins Byrne D. (2006). Comparison of ABTS, DPPH, FRAP, and ORAC assays for estimating antioxidant activity from guava fruit extracts. J. Food Compos. Anal..

[30] Zhang X.Y., Su J.Q., Wang X.Y., Wang X.Y., Liu R.X., Fu X., Li Y., Xue J.J., Li X.L., Zhang R. (2022). Preparation and properties of cyclodextrin inclusion complexes of hyperoside. Molecules.

[31] Deshaware S., Gupta S., Singhal R.S., Joshi M., Variyar P.S. (2018). Debittering of bitter gourd juice using beta-cyclodextrin: Mechanism and effect on antidiabetic potential. Food Chem..

[32] Iraklia M., Chatzopouloua P., Ekateriniadou L. (2018). Optimization of ultrasound-assisted extraction of phenolic compounds: Oleuropein, phenolic acids, phenolic alcohols and flavonoids from olive leaves and evaluation of its antioxidant activities. Ind. Crop. Prod..

[33] Wang B.X., Shen S.A., Qu J.P., Xu Z., Feng S.L., Chen T., Ding C.B. (2021). Optimizing total phenolic and oleuropein of Chinese olive (Olea europaea) leaves for enhancement of the phenols content and antioxidant activity. Agronomy.

[34] Yuan C., Du L., Jin Z.Y., Xu X.M. (2013). Storage stability and antioxidant activity of complex of astaxanthin with hydroxypropyl-beta-cyclodextrin. Carbohyd. Polym..

[35] Li S.J., Yue J.Z., Zhou W., Li L. (2015). An investigation into the preparation, characterization and antioxidant activity of puerarin/cyclodextrin inclusion complexes. J Incl. Phenom. Macrocycl. Chem..

[36] Zhu Z.Y., Luo Y., Liu Y., Wang X.T., Liu F., Guo M.Z., Wang Z., Liu A.J., Zhang Y.M. (2016). Inclusion of chrysin in beta-cyclodextrin and its biological activities. J. Drug Deliv. Sci. Technol..

[37] Sadaquat H., Akhtar M. (2020). Comparative effects of beta-cyclodextrin, HP-beta-cyclodextrin and SBE7-beta-cyclodextrin on the solubility and dissolution of docetaxel via inclusion complexation. J. Incl. Phenom. Macro..

[38] Batt D.K., Garala K.C. (2013). Preparation and evaluation of inclusion complexes of diacerein with beta-cyclodextrin and hydroxypropyl beta-cyclodextrin. J. Incl. Phenom. Macro..

[39] Zhang X.W., Zhang T.P., Lan Y.L., Wu B.J., Shi Z.H. (2016). Nanosuspensions containing oridonin/HP-beta-cyclodextrin inclusion complexes for oral bioavailability enhancement via improved dissolution and permeability. AAPS Pharmscitech.

[40] Hu Q., Fu X.L., Su Y.P., Wang Y.F., Gao S.H., Wang X.Q., Xu Y., Yu C.X. (2021). Enhanced oral bioavailability of koumine by complexation with hydroxypropyl-beta-cyclodextrin: Preparation, optimization, ex vivo and in vivo characterization. Drug Deliv..

[41] Zhang Y., Wang Q.S., Cui Y.L., Meng F.C., Lin K.M. (2012). Changes in the intestinal absorption mechanism of icariin in the nanocavities of cyclodextrins. Int. J. Nanomed..

[42] Li J., Geng S., Wang Y., Lv Y., Wang H., Liu B., Liang G. (2019). The interaction mechanism of oligopeptides containing aromatic rings with beta-cyclodextrin and its derivatives. Food Chem..

[43] Jing P., Ye T., Shi H., Sheng Y., Slavin M., Gao B., Liu L., Yu L.L. (2012). Antioxidant properties and phytochemical composition of China-grown pomegranate seeds. Food Chem..

[44] Yao Q., Shen Y., Bu L., Yang P., Xu Z., Guo X. (2019). Ultrasound-assisted aqueous extraction of total flavonoids and hy-droxytyrosol from olive leaves optimized by response surface methodology. Prep. Biochem. Biotechnol..

[45] Yang P., Luo J.H., Yan S., Li X.H., Yao Q. (2022). Permeation of hydroxypropyl-beta-cyclodextrin and its inclusion complex through mouse small intestine determined by spectrophotometry. Curr. Pharm. Anal..

